# Antimicrobial effect of silver-impregnated cellulose: potential for antimicrobial therapy

**DOI:** 10.1186/1754-1611-3-20

**Published:** 2009-12-04

**Authors:** Juyoung Kim, Soonjo Kwon, Erik Ostler

**Affiliations:** 1 Department of Biological Engineering, Utah State University, 4105 Old Main Hill, Logan, UT 84322, USA; 2 Ag Biotech LLC, 1704 Cordell Drive, Tallahassee, FL 32303, USA

## Abstract

**Background:**

Silver has long been known to have antimicrobial activity. To incorporate this property into multiple applications, a silver-impregnated cellulose (SIC) with low cytotoxicity to human cells was developed. SIC differs from other silver treatment methods in that the leaching of silver particles is non-existent and the release of ionic silver is highly controlled.

**Results:**

*Candida albicans*, *Micrococcus luteu, Pseudomonas putida*, and *Escherichia coli *were used for antimicrobial testing. No microbial cells were able to grow in the presence of SIC at concentrations above 0.0035 Ag w/v %. Even at a concentration of 0.00035 Ag w/v %, *P. putida *and *M. luteu *failed to grow, and *C. albicans *and *E. coli *exhibited diminished growth. To determine the cytotoxic effect of silver on human cells, five different concentrations of SIC were tested on human fibroblasts. In SIC concentrations of 0.035 Ag w/v % and below, no cytotoxicity was observed.

**Conclusion:**

The optimal concentration of SIC for a broad range of anti-microbial activity and low or negligible cytotoxicity was 0.0035 Ag w/v %. Although the highly controlled releasing characteristics of SIC would prove a substantial improvement over current technologies, further investigation for genotoxicity and other biocompatibility test will be required.

## Introduction

Silver in various forms has been used for centuries as an antimicrobial agent. It is reported that the ancient Greeks used silver coins as a way of disinfecting stored liquids [1]. In the late 1800's, silver nitrate solutions were used to prevent ocular infections in neonates. These same solutions were also found to be effective against typhoid and anthrax bacilli [2]. Colloidal silver was approved by the FDA for use in wound treatment in 1920 and through the first half of the 20^th ^century was used in applications such as decreasing the rubor in non-healing wounds and controlling infection in burn wounds [2]. Around the Second World War and with the advent of sulfonamide drugs and heavier use of antibiotics, the use of silver decreased. Approximately 30 years later, silver began to receive renewed clinical attention [3]. Precious metals, including silver, gold, and platinum, have the lowest side effects when in contact with the skin of humans and animals. Of these, silver has been used in a variety of applications such as treating skin diseases and other medical services because silver has antimicrobial activity and is not harmful to human beings. In order to employ the antimicrobial activity of silver, antimicrobial fibers, which were obtained by physically binding silver to cellulose, latex, polyethylene, polypropylene and the like, have been widely used in the field of the medical services [4-6].

The characteristics of silver which contribute to its scientific interest are as follows. First, yeasts, fungi, viruses, and a broad spectrum of anaerobic, aerobic, Gram-positive, and Gram-negative bacteria are susceptible to the effects of ionic silver [2,3,7,8]. As an advantage for its use in clinical applications, silver has also been categorized to be "oligodynamic", in that large bactericidal effects can be achieved by very small quantities [8-10]. In the active, soluble form as Ag^+ ^or Ag^0 ^[8], a proposed mechanism of action in bacteria is to interfere with DNA replication, inhibit electron transport, and/or cause conformational changes on the cell membrane [11]. Second, when used in proper concentrations, the amount of silver necessary to exhibit antimicrobial activity is, for the most part, not toxic to mammalian cells [12-14]. Third, bacteria has a relatively low propensity to develop a tolerance to silver [8]. The most important, recently used silver compounds include silver sulfadiazine (AgSD), metallic silver, silver acetate, and silver proteins. The silver compounds are used to prevent the infection in burn regions and ocular diseases and to eradicate warts, but some types of silver have had problems associated with cytotoxicity. In recent years, bandages have been treated with silver nitrate to obtain silver-engrafted bandages. However, the silver-engrafted bandages still have problems associated with the cytotoxicity to human beings [15-17] and silver leaching [18-20].

Cellulose, which is a common wound dressing, has also been impregnated with silver by a variety of methods. One method impregnated cellulose with silver nanoparticles by reducing silver nitrate (AgNO_3_) with sodium borohydride (NaBH_4_). The nanoparticles formed small spheres that were evenly adsorbed throughout the cellulose. When tested against *Escherichia coli *and *Staphylococcus aureus*, the antimicrobial efficacy was found to be more than 99.99% [21]. Another method used cellulose acetate (CA) that was produced by electrospinning fibers with AgNO_3_. The CA nanofibers were then irradiated by UV light at 245 nm to produce Ag nanoparticles [22]. Similar steps were followed to produce silver nanoparticles in CA, polyacrylonitrile (PAN) and polyvinyl chloride (PVC) matrices for testing antimicrobial properties [23]. Although these products do have varying degrees of antimicrobial efficacy, the continued development of products with maximized antimicrobial properties, good biocompatibility, and low cytotoxicity is a priority.

As described above, silver must be permanently engrafted into cellulose. The technology of permanently fixing silver in cellulose can be applied to fibers of medical wool or cotton, and is useful in a large number of applications including wood preservation, antibiotic bandages, water purification, and others [24-26]. In this study, we demonstrate a method of making silver-impregnated cellulose (SIC), comprised of associating ionic silver with water soluble polymers, thus introducing the Ag+-polymer complex into the cellulose matrix. *Candida albicans*, *Micrococcus luteus, Pseudomonas putida*, and *E. coli *were used for antimicrobial testing. To determine the effects of silver on cytotoxicity to human cells, five different concentrations of SIC were tested on human fibroblasts.

## Materials and methods

### Analytical Methods

Qualitative elemental analysis was conducted using Inductively Coupled Plasma (ICP, Perkin-Elmer Optima 3000 DV). The ICP was set to read silver at an optimized wavelength of 328.068 nm, with a relative detection limit of 0.9 ppb Ag. The method of digestion was as follows: an SIC pellet was shaken in 50 mL of deionized water at 150 rpm for 24 hours. The supernatant was tested via ICP for residual silver concentration. The pellet was then immersed in a simple digestion apparatus containing a boiling solution of nitric acid and hydrogen peroxide and left for 24 hours. The resulting solution of the digested pellet sample was diluted to 50 mL and tested via ICP for residual silver concentration. Concentration of silver, weight of media, and total weight of silver in media were measured as follows, and the results are listed in Table 1.

**Table 1:** Silver leached by cotton pellets.

Medium	Concentration of silver (mg/L)	Weight of medium (g)	Total weight of silver in media (mg/g)	
			after leaching	Control
Cotton pellet	6.846	0.7202	0.4752	0.4862

*The ICP was set to read silver at an optimized wavelength of 328.068 nm, with a relative LOD of 0.9 ppb Ag

#### Concentration of silver

Each medium was dipped in 25 mL of concentrated nitric acid (HNO_3_) and 25 mL of hydrogen peroxide solution (H_2_O_2_) respectively. Small glass beads were added to the solution to increase the boiling point and disperse heat. Each solution was heated to a temperature at which bubbles generated in an initial reaction did not enter the cooling machine, and then these were additionally heated for 24 hours. The solid media were completely solubilized (digested), and a concentration of silver was measured using ICP-MS.

#### Weight of media

Before beginning the experiments, the media samples were dried at 105°C for 2 hours in a desiccator, and cooled to room temperature. Then, dry weights of the media samples were measured.

#### Total weight of silver in media

The media were digested by a nitric acid and hydrogen peroxide solution. After digestion, each of the solutions was evaporated and distilled water was added to each of the solutions to compensate for the evaporated liquid, bringing the final volume back to 50 mL. Then, the concentration (mg/L) of silver in solution used for coating was converted to an amount (mg) of silver, and the amount (mg) of silver was then divided by the weight of the medium. This may be represented by the following Equation.

To analyze the characteristics of silver coated cotton (cellulose matrix), an Electroscan Environmental Scanning Electron Microscope (ESEM, Model E3) was used. Samples for ESEM were dried for at least a day at 103-105°C in a drying oven, and then cooled in a desiccator. Sputter coating with gold were performed to produce high topographic resolution in a cooled desiccator.

### Silver Coating Method

AgCl was dissolved in a reaction with NH_4_OH, and the dissolved silver ions then bound to a cellulose matrix. The NH_4_OH was released in the gaseous form of NH_3 _and a neutral pH was maintained throughout the reaction. AgCl in the amounts of 5, 0.5, 0.05, 0.005 and 0.0005 g were dissolved in 1 liter of NH_4_OH solution under the atmospheric conditions to produce final concentrations of 3.75, 0.75, 0.35, 0.035, 0.0035 and 0.00035 Ag w/v %, respectively. Oral cotton pellets (natural cotton pellets slightly coated with sodium carboxymethyl cellulose (Na-CMC)) were then added to the resulting solutions to allow the cellulose matrix to react with the silver, thus to form cellulose-OAg complexes. These reactions were carried out for 24 hours. Upon completion, the cellulose-OAg complexes were washed three times with water, and then dried. The final pH levels of the cellulose-OAg complexes were within a range of 6 to 8. All reactions were carried out at room temperature, and the drying temperature did not exceed 105°C.

### Microbial Cell Culture

The *C. albicans*, *M. luteus*, *P. putida*, and *E. coli *cultures were obtained from American Type Culture Collection (ATCC). All cell lines were cultivated in Luria Broth (LB) media for 24 hours at 37°C. Cells were spun down and washed twice in phosphate buffered saline (PBS) before being re-suspended in PBS. Two cell solutions of approximately 10^4 ^and 10^9 ^cells/mL were prepared and incubated in shaker flasks with silver-coated cotton pellets at six different concentrations. Starting concentration of microbial cells was ~10^4 ^or ~10^9 ^cells/mL. The effect of silver on the decrease in microbial cells was monitored by the optical density (OD_600_), measured with spectrophotometer (600 nm). After incubating microbial cells with silver-coated cotton pellets for 48 hours, 100 μL samples were used to inoculate an agar plate. These plates were incubated at 37°C overnight.

### Human Cell Culture

Fibroblasts originating from human skin were obtained from ATCC (CCD-25Sk, CRL-1474). Each well of 24 well plates contained six different concentrations of silver-coated cotton fibers (teared cotton pellets). Silver-coated cotton pellets were teared and distributed on the surface of each culture well. The fibroblasts were seeded at 2 × 10^4 ^cells/cm^2^onto the cotton fibers in 24 well plates. Fibroblasts were cultured in 1.0 mL of minimum essential medium (MEM) supplemented with non-essential amino acids, sodium pyruvate (1 mM), 10% fetal bovine serum (FBS), lactalbumin hydrolysate (0.1%), amphoterincin B (5.6 mg/L), and penicillin-streptomycin (100 U/mL). Six concentrations of silver-coated cotton fibers were used to determine the effects of silver on the human fibroblasts.

### Cell Viability

LIVE/DEAD assay kit (Molecular Probes, Eugene, OR) was used to identify the effect of silver-coated cotton fibers on cell viability and cytotoxicity.

## Results

### Structure of silver-polymer complex with the cellulose matrix

**Figure 1:**
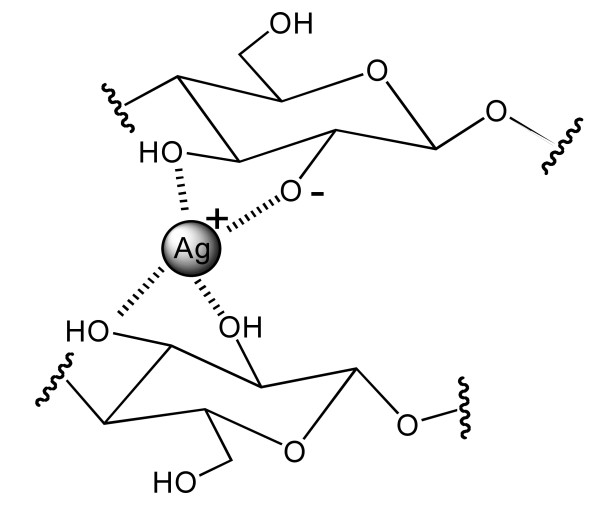
**Possible conformation of the compound formed by a cellulose radical binding to silver ions**.

The method for preparing a natural cellulose matrix bonded with silver is comprised of two steps: 1) introducing silver into a natural cellulose matrix by dipping the matrix in a solution of AgCl-dissolved in NH_4_OH; 2) and substituting a hydrogen (H) from a hydroxyl (-OH) group in cellulose with Ag by drying the silver ion-engrafted cellulose matrix, thus forming a cellulose-OAg complex having a chemical or a physical bond between the cellulose and silver molecules. AgCl, the source of silver for this reaction, reacts according to Equation 1 when dissolved in NH_4_OH. H+ was generally released from the cellulose hydroxyl group at the C-3 position because it had the highest acidity, causing the formation of an alkoxylate radical (R-O^-^). The cellulose radical (R-O^-^) was reacted with silver ions chemically binding the two components and forming a compound with a possible conformation that illustrated in Figure 1.(1)

**Figure 2:**
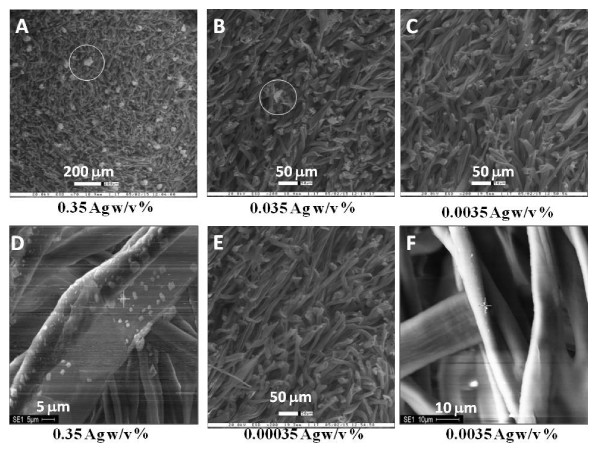
**ESEM picture of silver-coated cotton**. Samples for ESEM were dried for at least a day at 103-105°C in a drying oven, and then cooled in a desiccator. Sputter coating with gold were performed to produce high topographic resolution in a cooled desiccator. Circle indicated silver particles.

One silver ion may have up to four ligands, and hydroxyl groups in positions C-2 and C-3 of cellulose may bind to metal ions in a bidentate form. The silver bound in the alkoxylate form at the C-3 position and the interactions between the other hydroxyl groups cause the formation of a chelate compound, which prevents the metal ions from being leached in aqueous solutions. The cellulose-OAg complex represents a chemical bond between silver and the cellulose matrix. In addition to the chemical bonds, the silver physically binds to the cellulose, that is, silver is present in cellulose in the form of Ag_2_O, Ag and AgCl. The chemical binding of silver to cellulose to form the cellulose-OAg complex occured with a silver concentration of 0.0035 Ag w/v% in NH_4_OH solution. If the concentration of silver was below 0.0035 w/v%, no detectable physical binding of the silver was observed (See Figure 2). When the silver impregnated cellulose pellets are re-dissolved in water, no detectable amount of silver particle was leached from the cellulose-OAg complex. If the concentration of silver was below 0.0035 Ag w/v%, detectable amount of silver by ICP was the amount of the silver chemically bonded to cotton (cellulose matrix).

### Leaching test and digestion results of silver-coated cottonpellets

Concentration of silver, weight of media, and total weight of silver in media were measured, and the results are listed in Table 1. Data listed in Table 1 specify that the weights of silver in the cotton pellets measured before and after leaching fall within the error range of the analysis. These results revealed that the silver that chemically reacted with the natural cellulose according this method did not leach when the natural cellulose was re-dissolved in water.

### Anti-microbial effects of silver-coated cotton pellets

Following incubation with five different concentrations of SICs, testing samples were prepared and plated as described in 'Materials and Methods'. *M. luteus *and *P. putida *were not detected in samples cultured with a cotton pellet containing any concentration of silver. *C. albicans *and *E. coli *were only detected in the samples cultured with a pellet prepared in the solution having 0.00035 Ag w/v %, but were detected in reduced numbers (Table 2). Although not listed in Table 2, the microorganisms were not detected inside the pellet prepared in the solution having more than 0.0035 w/v % of silver.

**Table 2:** Antimicrobial effect of various concentrations of silver impregnated cellulose.

Microorganisms	Concentration of silver (w/v %)					
	
	0	0.00035	0.0035	0.035	0.35	0.7
*Candida albicans*	confluent	5% confluent	0	0	0	0
*Micrococcus luteus*	confluent	0	0	0	0	0
*Pseudomonas putida*	confluent	0	0	0	0	0
*Escherichia coli*	confluent	5% confluent	0	0	0	0

(Unit: CFU)

### Effect of silver-coated cotton fibers on human fibroblasts

**Figure 3:**
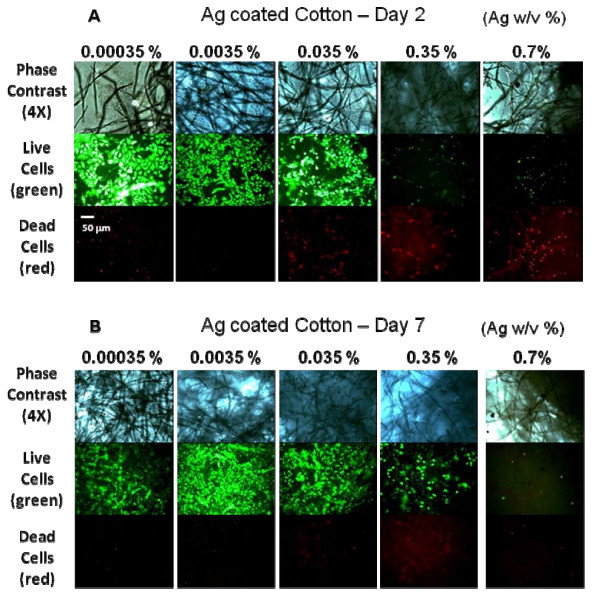
**Effect of silver-coated cotton fibers on cytoxicity to human fibroblasts**. Phase image (4X, top), viable cells (green, middle), and dead cells (red, bottom) were shown on silver-coated cotton fibers at day 2 (A) and day 7 (B). Each well of 24 well plates contained six concentrations of silver-coated cotton fibers (teared cotton pellets). Silver-coated cotton pellets were teared and distributed on the surface of each culture well. The fibroblasts were seeded at 2 × 10^4 ^cells/cm^2^ onto the cotton fibers in 24 well plates.

Human fibroblasts were allowed to grow on SIC as seen in Figure 3. Cells were attached to both the plastic surface and the fiber-like structure of the SIC. The effect of the silver-coated cotton fibers on cell viability and cytotoxicity was monitored at day 1, day 2, and day 7. Five different concentrations of SIC were tested with human fibroblasts. Viable cells (green) were predominant in SIC of 0.035 Ag w/v % or lower, and an increased portion of dead cells (red) were detected in SIC of 0.35 Ag w/v % or higher, at both day 2 and day 7. Therefore, it is apparent no cytotoxicity to human fibroblasts was observed in SIC concentrations of 0.035 Ag w/v % or lower (Figure 3).

## Discussion

Silver is preferably supplied in the form of AgCl, which is used as the silver source, and NH_4_OH is preferably used as the solvent. When NH_4_OH reacts with AgCl, other residual compounds are not formed except for NH_4_Cl, which is not harmful to humans. In this study, natural cellulose was used as the cellulose matrix. This cellulose is nontoxic and biodegradable, and has a huge pelletized surface area, making it ideal for environmental and medical applications. Cellulose may be obtained from various sources including both woody plants and non-woody plants, but the present invention is not particularly limited thereto. The cellulose-OAg complex is formed by the chemical binding of silver to the cellulose matrix. As a result, the cellulose-OAg complex is endowed with antimicrobial activity. The lowest concentration of silver in the matrix, which is sufficient for showing antimicrobial activity, is approximately 0.01 mg/g. This antimicrobial activity may be achieved using an NH_4_OH solution in which silver is present in a concentration of 0.00035 w/v% or more. When the silver concentration in the NH_4_OH solution is below this range, the silver chemically or physically bound to the cellulose matrix does not provide sufficient antimicrobial activity. The antimicrobial activity is improved with an increased concentration of silver. However, exceeding a concentration 7.5 Ag w/v% is economically undesirable because of the limited solubility of AgCl in the NH_4_OH solution. When silver is present in excessive concentrations, it may be toxic to human cells. Therefore, the silver concentration should not exceed approximately 0.35 Ag w/v %, at which point the material begins to show cellular toxicity to the human cells. The Food and Drug Administration (FDA) has established classifications for approximately 1,700 different generic types of devices and grouped them into 16 medical specialties referred to as panels. Each of these generic types of devices is assigned to one of three regulatory classes based on the level of control necessary to assure the safety and effectiveness of the device. The three classes and the requirements which apply to them are: Class I (General Controls), Class II (General Controls and Special Controls), and Class III (General Controls and Premarket Approval). General Controls are the baseline requirements of the FDA Food, Drug and Cosmetic (FD&C) Act that apply to all medical devices, Class I, II, and III. To use silver coated cotton as a medical device, test for *in vitro *cytotoxicity is initially required. Our *in vitro *cytotoxicity test meets the requirement of the test as per International Organization for Standardization (ISO 10993-5 Biological Evaluation of medical devices - Part 5: Tests for *in vitro *cytotoxicity, Reference Number ISO 10993-5:2009).

The status of binding (chemical or physical) between the cellulose and silver was shown in ESEM photographs (Figure 2). When the silver solution was used in concentrations of 0.035 to 0.35 w/v %, silver crystals was formed on the surface of cellulose (cotton). However, no detectable physical binding of the silver was observed in concentrations of 0.0035 w/v % or less. The silver crystals had a hexagonal shape and their size was approximately 1 to 10 μm in diameter. If the silver crystals were brought in contact with the human body, these crystals could possibly enter the blood stream and accumulate in blood vessels having a diameter of approximately 20 μm. The components were analyzed using an energy dispersive x-ray analysis system (EDS). The distribution of each component obtained from the x-ray analysis system was displayed on the map, and concentrations of the components were derived from an analysis of the EDS-Map (Data not shown). The analysis showed that the chemically bonded ionic silver was uniformly distributed over a surface of the cellulose matrix when the NH_4_OH solution including 0.0035 Ag w/v % or less was used. Also, silver particles were not detected in a leaching test using water as a solvent. These results revealed that silver chemically binds to cellulose by means of the mechanism represented by Equation 1. But we cannot exclude that silver ion still can be released from the cellulose-OAg complex.

The mechanism of the antimicrobial action of silver ions is not well characterized yet, but is closely related to their interaction with thiol groups [10,27-29], although other target sites remain a possibility [30,31]. These and other findings imply that interaction of Ag^+ ^with thiol groups in enzymes and proteins plays an essential role in bacterial inactivation, although other cellular components may be involved. Hydrogen bonding, the effects of hydrogen bond-breaking agents, and the specificity of Ag^+ ^for thiol groups were discussed in greater detail by Russell and Hugo [32]. Virucidal properties might also be explained by binding to thiol groups [33]. Lukens proposed that silver salts and other heavy metals such as copper act by binding to key functional groups of fungal enzymes [34]. Ag^+ ^causes the release of K^+ ^ions from microorganisms and the microbial cytoplasmic membrane is are associated with many important enzymes and is an important target site for Ag^+ ^activity [29,35,36]. In addition to its effects on enzymes, Ag^+ ^produces other changes in microorganisms. Silver nitrate causes marked inhibition of *Cryptococcus neoformans *growth and is deposited in the vacuole and cell wall as granules [37]. Ag^+ ^also inhibits cell division and damages the cell envelope and contents of *Pseudomonas aeruginosa *[38]. Bacterial cells increase in size, and the cytoplasmic membrane, cytoplasmic contents, and outer cell layers all exhibit structural abnormalities, although without any blebs (protuberances) [38].

The question that puzzles researchers most is how silver ions exhibit lower cytotoxicity to human cells, while showing significant anti-microbial effects. The answer may lie in the difference in membrane structure and enzyme properties between human cells and bacterial cells. It may also depend on the concentration of ionic silver and the structure of silver-polymer complex with cellulose. Furthermore, SIC, which has no particle leaching problems and no particle formation during molecular coating within a defined concentration range, has increased beneficial effects on human cells with increased antimicrobial activity.

## Competing interests

Ag Biotech LLC provided the experimental materials including silver-impregnated cellulose and supported publication costs. 'Method for preparing antimicrobial cotton of cellulose matrix having chemically and/or physically boded silver and antimicrobial cotton prepared therefrom' was disclosed by Juyoung Kim in Ag Biotech LLC.

## Authors' contributions

JK developed silver-impregnated cellulose and characterized materials. SK performed experimental design and data analysis for antimicrobial activities and cytotoxicty to human skin fibroblasts. EO assisted in data analysis. All authors have read and approved the final manuscript.
